# A Pilot Study on the Anti-Inflammatory Effects of Tacrolimus in Ankylosing Spondylitis: Evidence from Human Samples and a Murine Model

**DOI:** 10.5152/ArchRheumatol.2025.11154

**Published:** 2025-06-23

**Authors:** Sooin Park, Moon-Ju Kim, Yu Jeong Lee, Sung Min Yu, Hae-in Lee, So-Hee Jin, A-ra Choi, Seung Cheol Shim, Eun Jeong Won, Tae-Jong Kim

**Affiliations:** 1 Department of Rheumatology, Chonnam National University Medical School, Gwangju, Republic of Korea; 2 Department of Biomedical Sciences, Graduate School of Chonnam National University, Gwangju, Republic of Korea; 3 Division of Biodurg Evaluation, NDDC, Osong Medical Innovation Foundation (KBIOHealth), Cheongju, Republic of Korea; 4 College of Nursing, Nambu University, Gwangju, Republic of Korea; 5 Division of Rheumatology, Daejeon Rheumatoid and Degenerative Arthritis Center, Chungnam National University Hospital, Daejeon, Republic of Korea; 6 Department of Laboratory Medicine, Asan Medical Center, University of Ulsan College of Medicine, Seoul, Republic of Korea

**Keywords:** Ankylosing spondylitis, anti-inflammatory effects, tacrolimus

## Abstract

**Background/Aims:**

This study aimed to evaluate the potential anti-inflammatory and therapeutic effects of tacrolimus in ankylosing spondylitis (AS).

**Materials and Methods:**

Peripheral blood mononuclear cells (PBMCs) and synovial fluid mononuclear cells (SFMCs) from AS patients were treated with tacrolimus and analyzed via flow cytometry to measure inflammatory cytokine-producing cells (IFN-γ, IL-17A, and GM-CSF). Additionally, cytokine levels (IFN-γ, IL-17A, TNF-α, and GM-CSF) in ex vivo cultured PBMC supernatants were quantified using enzyme-linked immunosorbent assay (ELISA). The in vivo effects of tacrolimus were assessed in an AS mouse model by evaluating clinical arthritis scores and analyzing inflammatory cytokine-producing cells (IFN-γ, IL-17A, and TNF-α) via flow cytometry.

**Results:**

Tacrolimus significantly suppressed the production of inflammatory cytokines (IFN-γ, IL-17A, and GM-CSF) in PBMCs and SFMCs from AS patients. Cytokine levels (IFN-γ, IL-17A, TNF-α, and GM-CSF) in ex vivo PBMC cultures were also markedly reduced with tacrolimus treatment. In the AS mouse model, tacrolimus treatment resulted in significantly lower clinical arthritis scores and reduced production of inflammatory cytokines (IFN-γ, IL-17A, and TNF-α).

**Conclusion:**

Tacrolimus demonstrates potential as a therapeutic agent for AS by suppressing inflammatory cytokine production in PBMCs and SFMCs from AS patients and exhibiting anti-inflammatory effects in an arthritis mouse model.

## Introduction

**Main Points**

- Tacrolimus significantly reduced inflammatory cytokine production in both human immune cells and the AS mouse model.
- Clinical arthritis severity was significantly alleviated in vivo.
- Tacrolimus may serve as a promising therapeutic agent for AS by modulating pathogenic cytokine responses.

Ankylosing spondylitis (AS) is a chronic, progressive inflammatory arthritis that primarily targets the axial skeleton and sacroiliac joints, representing a systemic inflammatory condition.^1,2^ A hallmark feature of AS is enthesitis, the inflammation of enthesial sites where tendons, ligaments, and joint capsules attach to bone. This inflammation often manifests as back pain and stiffness, progressively impairing joint mobility over time. Another distinctive feature of AS is the coexistence of new bone formation and bone resorption, which contributes to structural damage and ankylosis.^3^

Although the precise pathogenesis of AS remains incompletely understood, strong evidence points to the involvement of genetic predisposition, particularly the HLA-B*27 allele, and dysregulated immune responses involving the IL-23/IL-17A cytokine axis. These factors are thought to play critical roles in the development and progression of AS.^2,4-7^

Tacrolimus, a widely used immunosuppressant, is primarily utilized in the management of solid-organ transplantation to prevent graft rejection. Its mechanism of action involves the inhibition of calcineurin, leading to suppressed T-cell activation and proliferation.^8^ Beyond transplantation, tacrolimus has demonstrated effectiveness in treating T-cell-mediated inflammatory diseases, such as inflammatory bowel disease, by mitigating inflammation.^9^ In recent years, tacrolimus has also been employed in the treatment of rheumatoid arthritis (RA), where it has shown therapeutic benefits and is now recognized as an antirheumatic drug.^10^ However, reports on the efficacy of tacrolimus in AS remain sparse. Despite its known anti-inflammatory properties, there is limited evidence regarding its impact on the progression and disease course of AS.

Given tacrolimus’s inhibitory effects on the expression of pro-inflammatory cytokines such as IL-17A and tumor necrosis factor-α (TNF-α), primarily through the modulation of Th17 cell activity,^11^ and its established role in managing active RA,^12^ it was hypothesized that tacrolimus could similarly reduce inflammation in AS. Herein, the aim was to evaluate the potential anti-inflammatory effects of tacrolimus in the context of AS.

## Materials and Methods

### Human Samples

All patients selected for the experiment satisfied the modified New York criteria for AS. Peripheral blood mononuclear cells (PBMCs) were obtained from 6 patients, and synovial fluid mononuclear cells (SFMCs) were collected from the knee joints of the 6 patients with active disease. The demographic characteristics of the patients are shown in Table 1. This study was conducted in a Korean population, and all participants were of Asian race and Korean ethnic background. This study was carried out in compliance with the Helsinki Declaration. The Ethics Committee of Chonnam National University Bitgoeul Hospital approved this study (approval number: CNUBH-2023-020, date: September 5, 2023) and written informed consent was obtained from all subjects. 

### Experimental Mouse Model

All experiments were conducted with the approval of the Institutional Animal Care and Use Committee of the Institutional Animal Care and Use Committee (CNU IACUC-H-2023-20, date: June 29, 2023). To evaluate the effect of tacrolimus on arthritis in in vivo model, SKG ([Sakaguchi] mouse) mice on a BALB/c background were obtained from CLEA Japan (Tokyo, Japan) and bred in a specific pathogen-free facility. In this study, all the mice were female, and the mice were categorized into 3 groups: a normal control group (n = 6 mice), a disease control group (n = 7 mice), and a tacrolimus treatment group (n = 10 mice). Experimental arthritis was induced in 8-week-old mice in both the disease control and tacrolimus treatment groups by intraperitoneal (i.p.) injection of 3 mg/kg of curdlan suspension (Wako, Osaka, Japan).

One week after curdlan injection, tacrolimus was prepared by diluting in water and orally administered to the treatment group. Mice in the disease control group were given water, while the tacrolimus treatment group received 1 mg/kg per day of tacrolimus through their drinking water. Water and drug intake were monitored weekly to ensure consistent consumption across groups. Notably, no significant differences in water intake were observed between the disease control and tacrolimus treatment groups, confirming comparable administration conditions (Table 2).

The experiment lasted for 7 weeks. The mice’s clinical signs were monitored twice a week and scored by 2 independent observers. Scoring was performed by summing the affected joints, as described in a previous report:^13^ 0 = asymptomatic, 0.1 = Swelling per toe, 0.5 = swelling of the ankle, 1 = severe swelling of the ankle. Six points were the highest possible points.

### Flow Cytometry

To determine whether tacrolimus treatment suppresses inflammatory cytokines producing cells in a human setting, PBMCs and SFMCs were isolated from AS patients and the percentages of IFN-γ, IL-17A, and GM-CSF producing cells were analyzed. PBMCs and SFMCs were isolated and suspended in a complete medium (RPMI 1640, 2 mM L-glutamine, 100 units/ml of penicillin, and 100 mg/ml of streptomycin) supplemented with 10% fetal bovine serum (Gibco BRL, Grand Island, NY, USA), and then seeded into 96-well plates at a cell density of 1 × 10^6^ cells/well. Cells in a 96-well culture plate were treated with 1µg/mL of tacrolimus and then were activated with Dynabeads Human T-Activator CD3/CD28 (11131D, Gibco, USA) to obtain a bead-to-cell ratio of 1:1. Cells were then incubated in a humidified CO_2_ incubator at 37°C for 24 h. Cells were stained with Pacific Blue-conjugated anti-CD4 (300521, BioLegend, USA) and anti-Fixable Viability Dye-eFuor780 (65-0865-14, Invitrogen, USA). Cells were washed, fixed, permeabilized with Cytofix/Cytoperm buffer, and stained intracellularly with FITC Mouse anti-human IFN-γ (552887, BD, USA), APC-conjugated anti-IL-17A (512334, BioLegend, USA), and PerCP-Cy5.5-conjugated anti-GM-CSF (502312, BioLegend, USA) antibody.

Flow cytometry was used to analyze IFN-γ-, IL-17A-, and TNF-α-producing cells in splenocytes isolated from the spleens of SKG mice. Cell-surface markers and cytokine expression were detected by flow cytometry after stimulating with Brefeldin A (1 μL/mL), PMA (100 ng/mL), and ionomycin (1 μM) for 4 h. Cells were subjected to FITC anti-mouse IFN-γ Antibody (505806, Biolegend, CA), followed by PE anti-mouse IL-17A Antibody (506904, Biolegend, CA), APC anti-mouse CD4 Antibody (100412, Biolegend, CA), and APC/Cyanine7 anti-mouse TNF-α Antibody (506344, Biolegend, CA). The data were analyzed using FlowJo Software (BD, USA).

### Enzyme-Linked Immunosorbent Assay

To examine the levels of pro-inflammatory cytokines in the stimulated human PBMCs, ex vivo cultured supernatants from PBMCs were measured using ELISA assays. The kits used for the experiments are as follows, Human IFN-γ ELISA (88-7314, Invitrogen, Austria), Human IL-17A ELISA (88-7176, Invitrogen, Austria), Human TNF-α ELISA (88-7346, Invitrogen, Austria), and Human GM-CSF ELISA (88-8337, Invitrogen, Austria). The detection antibodies were added to the samples to specifically target the cytokines of interest, followed by horseradish peroxidase (HRP)-conjugated with streptavidin. The enzyme conjugate was then reacted with tetramethylbenzidine (TMB) solution, generating a fluorescent signal. The optical density (OD) was measured at 450 nm using a SpectraMax® M2 microplate reader (Molecular Devices Corp., USA). Finally, the data were analyzed by comparing the OD values to a standard curve generated from reconstituted cytokine standards.

### Statistical Analysis

Statistical analysis was performed using GraphPad Prism software version 10 (GraphPad, Inc.; San Diego, CA, USA). The statistical significance of differences between groups was assessed using a Mann–Whitney test. The Wilcoxon matched-pairs signed-rank test was used for paired samples and two-way ANOVA with multiple comparisons test were used for comparing 3 groups. For all graphs, *P* value less than .05 was considered significant and marked as follows: *P < .05; **P < .01; ***P < .001; and ****P < .0001.

## Results

### Tacrolimus Suppresses Pro-inflammatory Immune cells in Peripheral Blood Mononuclear Cells and Synovial Fluid Mononuclear Cells from Ankylosing Spondylitis Patients

**Figure 1.**
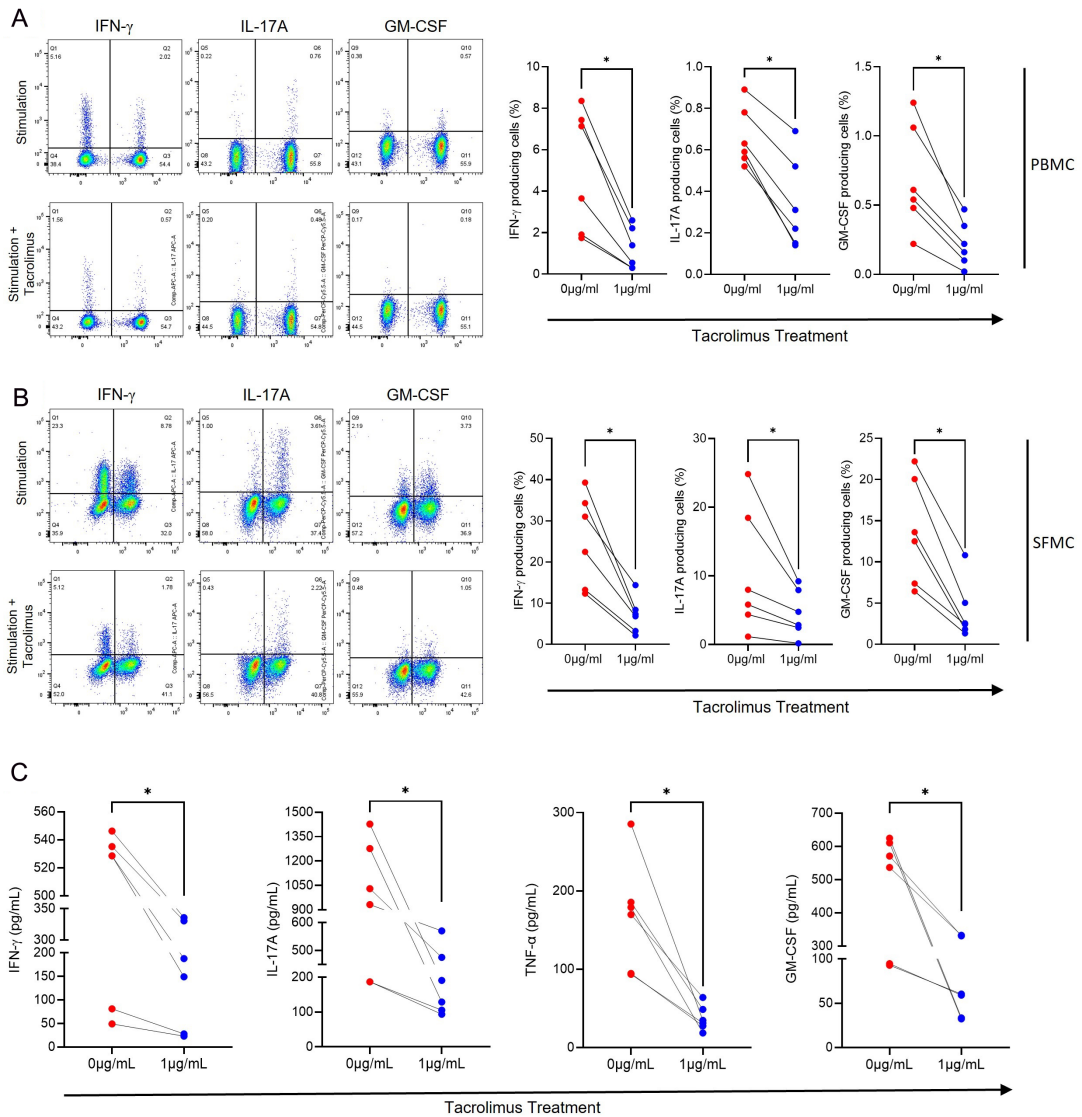
Tacrolimus treatment suppresses the production of inflammatory cytokines from PBMCs and SFMCs in patients with ankylosing spondylitis. Cells were activated with Dynabeads human CD3/CD28 in the presence or absence of tacrolimus for 24 hours. The cells were then incubated for 24 hours. Percentages of IFN-γ-, IL-17A-, and GM-CSF- producing cells were analyzed from (A) PBMCs and (B) SFMCs by flow cytometry. (C) In ex vivo cultured supernatants from the stimulated PBMCs, levels of IFN-γ, IL-17A, TNF-α, and GM-CSF were measured by ELISA. Symbols represent the individual sample. *P < .05 by Wilcoxon matched-pairs signed-rank test. ELISA, enzyme-linked immunosorbent assay; PBMCs, peripheral blood mononuclear cells; SFMCs, synovial fluid mononuclear cells.

We aimed to determine whether tacrolimus treatment suppresses IFN-γ-, IL-17A-, and GM-CSF-producing cells in peripheral blood and synovial fluid from AS patients. PBMCs and SFMCs from AS patients were stimulated and cultured ex vivo for 24 hours in the presence or absence of tacrolimus. The percentages of IFN-γ, IL-17A, and GM-CSF-producing cells among total lymphocytes were analyzed using flow cytometry. Treatment with tacrolimus (1 μg/mL) significantly decreased the frequencies of IFN-γ-, IL-17A-, and GM-CSF-producing cells in PBMCs compared to untreated cells (Figure 1A). Similarly, consistent results were observed in SFMCs, where the frequencies of IFN-γ-, IL-17A-, and GM-CSF-producing cells were also significantly reduced following tacrolimus treatment (Figure 1B). Additionally, the levels of IFN-γ, IL-17A, TNF-α, and GM-CSF in PBMC supernatants were analyzed using ELISA assays. Tacrolimus treatment markedly reduced the production of all these cytokines in the supernatants of ex vivo cultured PBMCs (Figure 1C).

### Tacrolimus Suppressed Arthritis in an In Vivo Model

**Figure 2.**
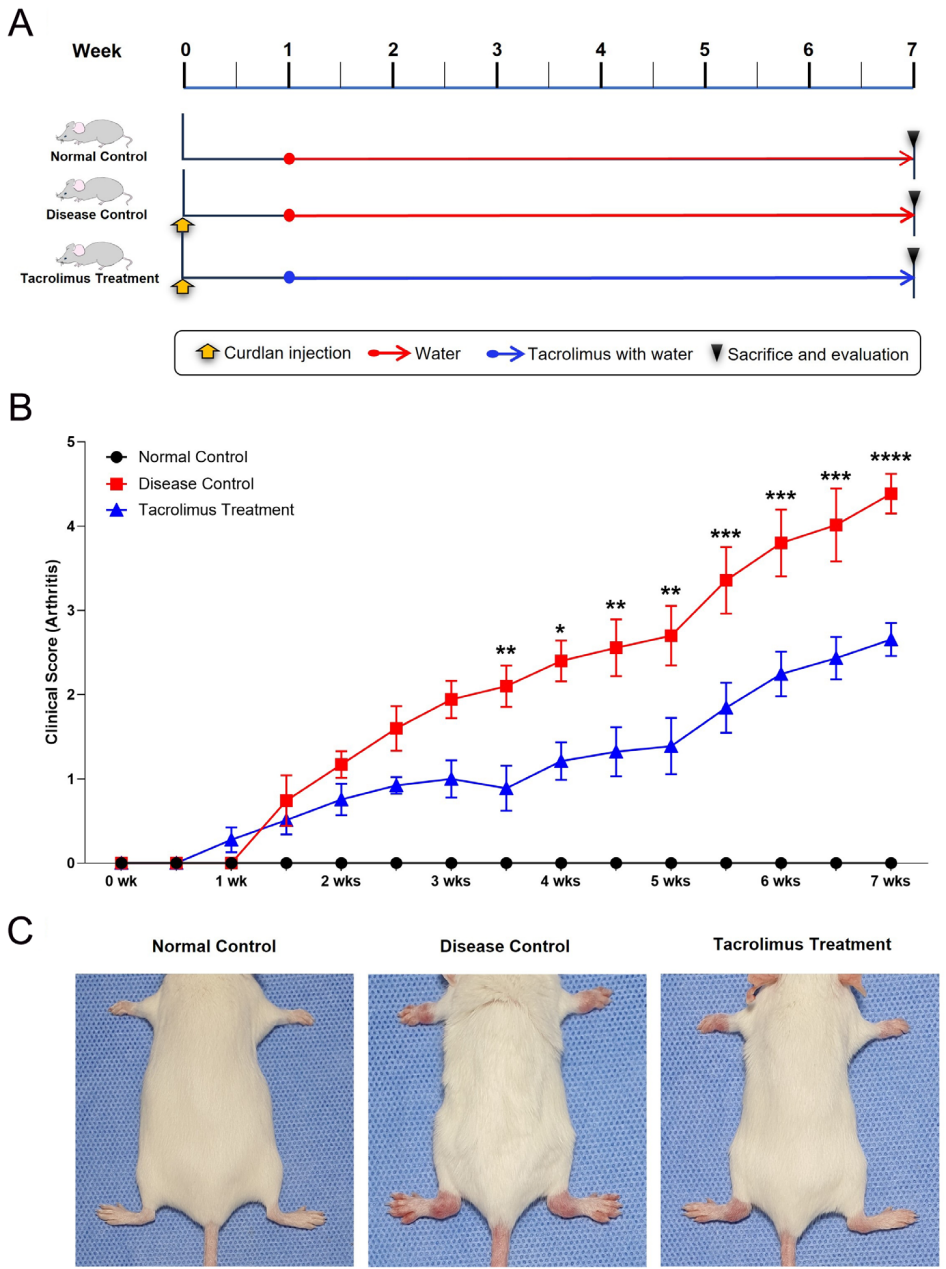
Tacrolimus reduces symptom severity in the SKG mouse model. (A) A diagram of how the experiment was designed. (B) The clinical scores for each group (normal control: n = 6 mice; disease control: n = 7 mice; tacrolimus treatment: n = 10 mice) were assessed over 7 weeks based on the severity of clinical arthritis signs. The values presented are the mean ± S.E.M. *P < .05, **P < .01, ***P < .001, ****P < .0001 by two-way ANOVA. (C) Representative images of SKG mice in each group at the end of the experiment are shown.

To investigate the effects of tacrolimus on arthritis, an experiment was conducted using SKG mice. Symptoms were induced in both the disease control and tacrolimus treatment groups by intraperitoneal injection of curdlan. One week after the curdlan injection, the mice were provided with either water (disease control group) or tacrolimus diluted in water (tacrolimus treatment group). After 7 weeks of experimentation, the mice were sacrificed, and symptom severity was evaluated based on clinical scores (Figure 2A). The clinical scores demonstrated that tacrolimus significantly reduced symptom severity starting from week 3.5 (2.1 ± 0.25 vs. 0.89 ± 0.27, *P* = .009) and continuing through week 7 (4.39 ± 0.23 vs. 2.66 ± 0.20, *P* < .0001) (Figure 2A). Representative images of SKG mice from each group further illustrated the findings. Mice in the tacrolimus treatment group exhibited reduced redness and swelling in the ankle joints compared to those in the disease control group (Figure 2C).

### Tacrolimus Exhibits Inhibitory Effects on Inflammatory Cytokine Production in Murine Splenocytes

**Figure 3.**
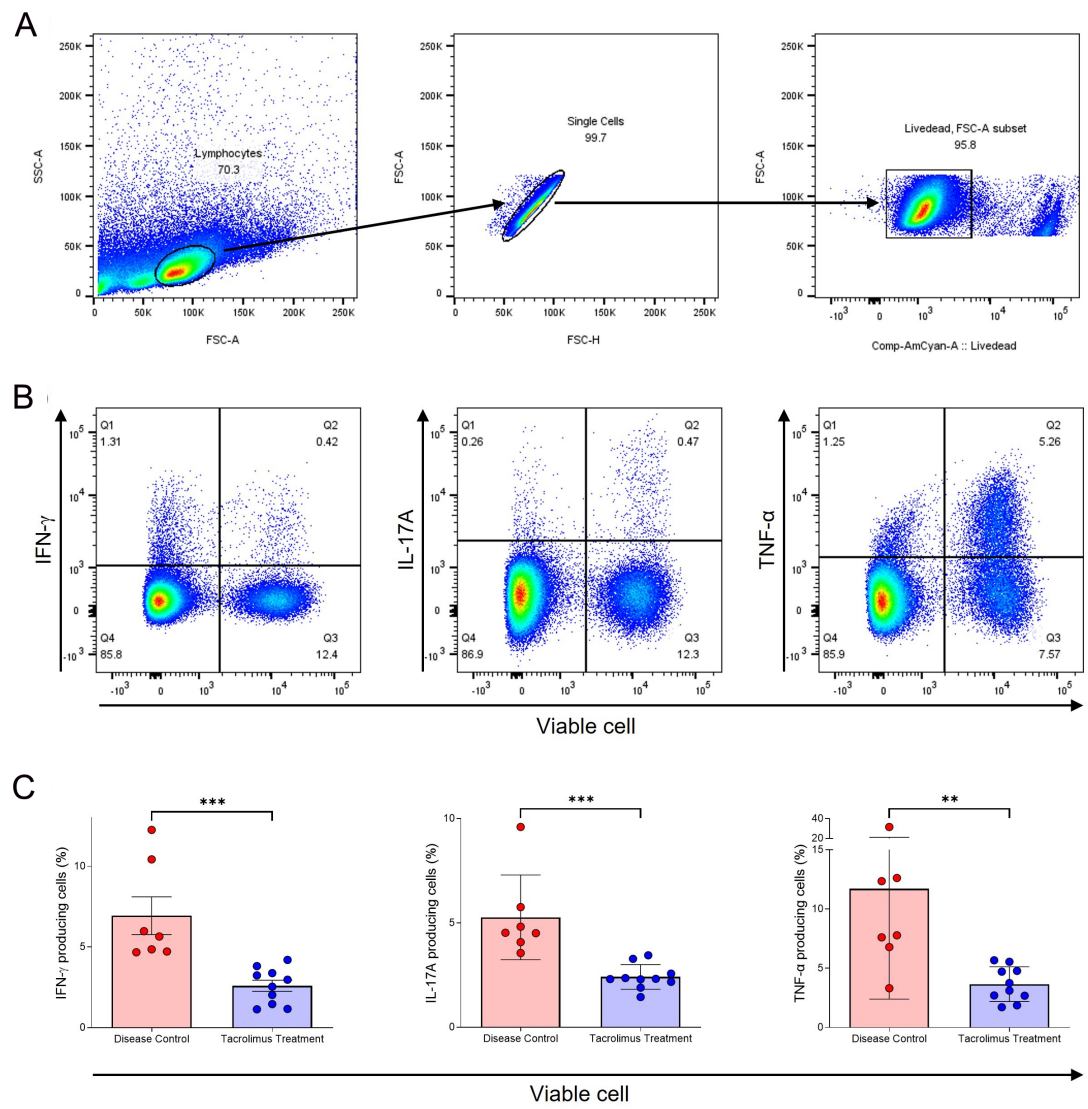
Tacrolimus suppresses the production of inflammatory cytokines in murine splenocytes. Flow cytometry was used to analyze IFN-γ-, IL-17A-, and TNF-α-producing cells from splenocytes of SKG mice in each group. Cells were stimulated with Brefeldin A (1 μL/mL), PMA (100 ng/mL), and ionomycin (1 μM) for 4 hours. (A) Schematic plots of the gating strategy are presented. (B) Representative images showing IFN-γ-, IL-17A-, and TNF-α-producing cells are presented. (C) Percentages of IFN-γ-, IL-17A-, and TNF-α-producing cells in each group were measured. Symbols represent the individual mice. ns: not significant, **P < 0.01, ***P < 0.001 by Mann-Whitney test.

Splenocytes were isolated from the spleens of SKG mice. The gating strategy used for flow cytometry analysis is illustrated. Lymphocytes were initially gated based on size and granularity using forward scatter (FSC) and side scatter (SSC) parameters, comprising 70.3% of the total splenocyte population. Viable cells (95.8%) were then identified using Comp-AmCyan-A Livedead fluorescent staining (Figure 3A). The percentages of IFN-γ, IL-17A, and TNF-α-producing cells within the viable cell population were measured across groups. Representative flow cytometry plots showing the populations of IFN-γ-, IL-17A-, and TNF-α-producing cells are presented (Figure 3B). The results revealed that the frequencies of IFN-γ-, IL-17A-, and TNF-α-producing cells from SKG splenocytes were significantly reduced in the tacrolimus treatment group compared to the disease control group. Specifically, tacrolimus-treated mice exhibited reduced frequencies of IFN-γ (6.93 ± 1.17 vs. 2.59 ± 0.35, *P *= .0010), IL-17A (5.27 ± 0.77 vs. 2.41 ± 0.19, *P* = .0006) and TNF-α (11.72 ± 3.52 vs. 3.66 ± 0.46, *P* = .0023) (Figure 3C).

## Discussion

In this study, it was found that tacrolimus exhibited significant anti-inflammatory effects in AS, as evidenced by data from both AS patients and a murine model. However, several limitations that may affect the generalizability of the findings were acknowledged. First, the sample size of AS patients was relatively small. Second, histological analysis of spinal bone inflammation in the SKG model could not be performed. Despite these limitations, all patient-derived samples consistently demonstrated that tacrolimus effectively suppressed the secretion of pro-inflammatory cytokines in PBMCs and SFMCs. Moreover, results from murine splenocytes further supported the anti-inflammatory effect of tacrolimus.

In experiments using PBMCs and SFMCs from AS patients, tacrolimus treatment markedly suppressed the secretion of pro-inflammatory cytokines, including IFN-γ, IL-17A, and GM-CSF. Similarly, in the mouse model, tacrolimus treatment resulted in significantly lower clinical arthritis scores compared to the disease control group. Furthermore, tacrolimus effectively reduced the frequencies of IFN-γ-, IL-17A-, and TNF-α-producing cells in splenocytes isolated from SKG mice. These findings suggest that tacrolimus may serve as a potential therapeutic option for AS by mitigating inflammatory responses.

Tacrolimus is a potent immunosuppressive agent widely used in the prevention and treatment of solid-organ transplant rejection. It exerts its effects by binding to intracellular proteins called immunophilins (FKBP) after entering T cells. This complex inhibits calcineurin phosphatase, an enzyme essential for the activation of the nuclear factor of activated T cells (NF-AT), a transcription factor required for the expression of cytokine genes in T cells. As a result, tacrolimus effectively suppresses T-cell activation and cytokine gene transcription.^14,15^ This mechanism underlies tacrolimus’s therapeutic effects in various autoimmune and inflammatory diseases. In autoimmune diseases like muscle-specific tyrosine kinase antibody-positive myasthenia gravis (MuSK-MG), tacrolimus has demonstrated the ability to suppress CD4 and CD8 T-cell proliferation and significantly reduce Th1 and Th17 responses. This includes decreased frequencies of IFN-γ-, IL-2-, and IL-17A-producing CD4 T cells, as well as IFN-γ- and IL-2-producing CD8 T cells.^16^ Tacrolimus is also widely used in lupus nephritis (LN), where it has shown non-inferiority to mycophenolate mofetil when combined with prednisolone in patients with biopsy-confirmed active LN.^17 ,18^ As Th17 cells and the cytokine IL-17A play a key role in LN pathogenesis,^19^ tacrolimus’s inhibitory effects on these pathways make it a compelling therapeutic option. In addition to systemic applications, tacrolimus is available as a topical treatment and is a first-line steroid-sparing immunomodulator for inflammatory chronic pruritus and atopic dermatitis.^20,21^ Tacrolimus is also a standard prophylactic agent for graft-versus-host disease when combined with methotrexate.^22^

In the context of axial spondyloarthritis (axSpA), genome-wide association studies have highlighted T cells as central to the disease’s pathogenesis.^23^ Ankylosing spondylitis, a subtype of axSpA, includes a spectrum of immune-mediated inflammatory diseases, such as reactive arthritis, psoriatic arthritis, and arthritis associated with inflammatory bowel disease.^24^ The strong association of HLA-B27 with AS further underscores the critical role of T cells in this condition.^25^ Given its T-cell-targeted mechanisms, tacrolimus holds the potential for therapeutic efficacy in reducing inflammation in AS. Moreover, tacrolimus has shown efficacy in related conditions, such as Crohn’s disease, where it suppresses IL-12/IL-23 p40, IL-6, and TNF-α production by activated macrophages. Tacrolimus has also been effective in treating fistulae refractory to anti-TNF-α therapies.^26^ These findings suggest that tacrolimus’s ability to suppress pro-inflammatory responses may be applicable to AS, particularly given its inhibitory effects on CD4 and CD8 T cells, Th17 responses, and cytokine expression. Additionally, tacrolimus has demonstrated the ability to inhibit IL-17A-induced osteoclastogenesis from human monocytes and to reduce the expression of IL-17A and TNF-α by decreasing the proportion of Th17 cells.^27^ These findings strongly support its potential therapeutic effects in AS, where Th17 cells play a pivotal role in disease development and progression.

It is noteworthy that the study first demonstrates a therapeutic effect of tacrolimus on AS. These findings suggest its potential as a therapeutic option for AS patients, warranting further clinical studies to expand its applications.

## Figures and Tables

**Table 1.** Clinical Characteristics of the Patients Enrolled in this study

Clinical Characteristics	AS	
	PBMCs	SFMCs
Total number	6	6
Age, mean ± SD (years)	27.17 ± 6.18	42.67 ± 20.02
Male, n (%)	5 (83.33)	6 (100)
Disease duration, mean ± SD (years)	1.84 ± 3.03	1.98 ± 2.08
BASDAI score, mean ± SD	3.64 ± 1.87	4.53 ± 1.26
AS-DAS, mean ± SD	3.5 ± 0.73	3.8 ± 0.54
Extra-musculoskeletal menifestation		
Uveitis, n (%)	0 (0.0)	0 (0.0)
Psoriasis, n (%)	0 (0.0)	0 (0.0)
Inflammatory bowel disease, n (%)	0 (0.0)	0 (0.0)
CRP, mean ± SD (mg/dl)	3.1 ± 2.86	3.88 ± 3.82
HLA-B27, n (%)	6 (100.0)	3 (50.0)
Recent treatments		
Naive, n (%)	1 (16.6)	1 (16.6)
NSAID use last 3 months, n (%)	5 (83.3)	4 (66.6)
Steroids use, n (%)	0 (0.0)	1 (16.6)
Biologic use, n (%)	0 (0.0)	4 (66.6)

AS-DAS, Ankylosing Spondylitis-Disease Activity Score; BASDAI, Bath Ankylosing Spondylitis Disease Activity Index; CRP, C-reactive protein; HLA, human leukocyte antigen; NSAID, non steroidal anti-inflammatory drug; PBMCs, peripheral blood mononuclear cells; SFMCs, synovial fluid mononuclear cells.

**Table 2.** Water Consumption in the Disease Control and Tacrolimus Treatment Groups

		Water Consumption (mL)		*P*
		Disease Control	Tacrolimus 1 mg/kg	
Week 1	Total	142.5	140.0	.874
	Per Mouse	4.07	4.0	.878
Week 2	Total	142.5	140.0	.874
	Per Mouse	4.1	4.0	.878
Week 3	Total	140.0	140.0	.999
	Per Mouse	4.0	4.0	
Week 4	Total	142.5	132.5	.626
	Per Mouse	4.1	3.8	.624
Week 5	Total	127.5	130.0	.500
	Per Mouse	3.6	3.7	
Week 6	Total	142.5	132.5	.500
	Per Mouse	4.1	3.8	
Week 7	Total	174.0	150.0	.426
	Per Mouse	4.4	3.8	.424

## Data Availability

The data that support the findings of this study are available on request from the corresponding author.
